# A scoping review of national policies for hierarchical medical system in China since the 2009 health reform

**DOI:** 10.3389/fpubh.2025.1606842

**Published:** 2025-07-09

**Authors:** Tingting Cai, Yuting Hua, Leyi Fang, Yunhui Xia, Jiantong Shen, Jianlin Lou

**Affiliations:** ^1^Medical School of Huzhou University, Huzhou, China; ^2^Huzhou Key Laboratory for Precision Prevention and Control of Major Chronic Diseases, Huzhou University, Huzhou, China

**Keywords:** China, health reform, hierarchical medical system, policy review, policy

## Abstract

**Background:**

Universal health coverage (UHC) is driving the global health agenda. In 2009, the Chinese government launched a new round of healthcare reform toward UHC, and made remarkable progress in UHC through the establishment of a hierarchical medical system (HMS). We aim to summarize and review the evolution of China’s hierarchical medical system policy.

**Methods:**

Eligible policies were identified between March 7, 2009 and December 31, 2022, by searching the official websites of the PKULAW Database, the Chinese State Council, and related ministries. Policy screening and data extraction were conducted by two researchers independently and discrepancies were resolved by consensus. Policy maker, policy initiatives, policy instrument, policy structure and evolution were visually analyzed. The people-centered integrated care model was used as the framework for analysis.

**Results:**

150 policy documents were included, most of which were issued in the 13th Five-Year Plan. There were 14 ministries involved in policy making, and they cooperated with each other, led by the State Council, with the National Health Commission and the National Administration of Traditional Chinese Medicine as the core. The pattern of policy making was top-down and bottom-up. Several areas showed to have strong policy support, including reforming the healthcare service delivery system, strengthening the primary health care workforce, reforming the health insurance system, and digitizing the health care system. Supply-side and environmental policy instruments were used the most while demand-side policy instruments were used the least.

**Conclusion:**

China’s hierarchical medical system had completed the transformation from local pilot to national promotion. Based on the results of the study, we propose the following recommendations: (1) a unified policy framework is necessary to coordinate the development and regulation of hierarchical medical system and facilitate multi-sectoral cooperation, (2) in-depth reforms in the key regions in hierarchical medical system, such as public hospital reform, medical insurance payment, (3) balance the use of various policy instruments, giving full consideration to the characteristics of hierarchical medical system at different stages.

## 1 Introduction

Universal health coverage (UHC) has been identified as a global healthcare priority, as an important element of the United Nations Sustainable Development Goals (SDGs) and WHO’s Thirteenth General Program of Work (GPW13) (1–4). UHC is based on the principle that all individuals and communities should receive the health services that they need without suffering financial hardship. In September 2019, all United Nations member states made a strong commitment to achieve UHC by 2030; as part of the global effort to build a healthier world for all (5). Aging population, urbanization, and unhealthy lifestyles make chronic noncommunicable diseases, mental illness, and injuries as the major disease burden (6–9). People’s demand for disease prevention, rehabilitation, and care services has increased, and the demand for health services has multilevel and diversified characteristics. However, due to the fragmentation of the health care system, weak coordination among health care providers, hospital-based and disease-centered curative care models, and neglect of health promotion and primary prevention, reduce the capacity of the health care system to provide universal, equitable, high-quality and financially sustainable care (10, 11). Universal health coverage will not be achieved without improvements in service delivery. In past decades, many countries pursuing universal coverage have relied on various approaches. The UK’s National Health Service model utilizes general taxation, a single risk pool, and publicly provided universal healthcare services (12, 13). By contrast, India, Indonesia, and Vietnam utilize general taxation to finance health coverage for low-income populations, while Nigeria employs general revenues derived from debt relief to fund pilot health insurance schemes targeting pregnant women and children (14). In China, the government launched a new round of health care reform toward UHC in 2009, and further proposed “Healthy China 2030.” Establishing a hierarchical medical system to overhaul the existing hospital-centric and treatment-based delivery system is an important part of China’s healthcare reform (15). In 2015, the Chinese government issued a guiding opinion on promoting the construction of a hierarchical medical system, proposing that by 2020, a hierarchical medical system will be basically established, in which health care facilities at each level (tertiary, secondary, and primary) deliver services according to their designated functions; care across the levels was to be coordinated with bidirectional referral mechanisms. Under the hierarchical model, patients are classified according to the severity of the disease and the difficulty of treatment, and patients of different conditions receive medical services from different levels of medical institutions. Thus, health-care facilities at each level provide appropriate, continuous, and high-quality services to patients according to their designated functions, which can effectively control the unreasonable growth of healthcare costs and promote equitable access to essential healthcare services (16). Under the series of policies issued by the Chinese government, the hierarchical medical system has been basically established, and significant progress has been made in improving the coverage of medical services and reducing inequalities (17). Health care reform and the construction of an integrated health care system are common challenges faced by health care systems in all countries. China’s practice and experience can provide valuable lessons and guidance for other countries, especially developing countries. To provide an in-depth analysis of the evolution of China’s hierarchical medical system policy and its development trends, we adopt a scoping review-based analytical framework to review profile of China’s hierarchical medical system policies (18). Specifically, the objectives of this review are to: first, to identify the volume and variety of available policies from 2009 to 2022; second, to explore the patterns of multi-sectoral collaboration in policy-making and the progress of key policy evolution; third, to identify key areas of China’s hierarchical medical system from the perspective of the health system; fourth, to identify the distribution of main policy instruments and their evolution.

## 2 Methods

### 2.1 Protocol and registration

This scoping review is reported in accordance with the PRISMA extension for Scoping Reviews (PRISMA-ScR, Appendix 1) (19). The protocol was registered prospectively in the Open Science Framework.^1^

### 2.2 Eligibility criteria

Eligible policy documents met the following criteria.

Inclusion criteria:

1. The policy document was issued between March 7, 2009, and December 31, 2022; and
2. The policy document was issued by the State Council or its affiliated ministries; and
3. The policy document was related to a hierarchical medical system; and
4. The types of policy documents were the Order, Opinion, Notice, and Notification (Appendix 2).

Exclusion criteria:

1. Any policy document published before March 7, 2009; or
2. The scope of the policy document was limited within a limited context, e.g., list, the statistical data, fact statement; or
3. The full text of the policy was not publicly available; or
4. The policy purpose was one-off projects; or
5. The policy was not substance about HMS, e.g., not associated with any domain of HMS.

We provide some questions used to facilitate policy identification in the search of websites (Appendix 3).

### 2.3 Information sources

A search was conducted through the publicly accessible official websites of the PKULAW Database,^2^ the Chinese State Council, and its affiliated ministries. The inclusion of affiliated ministries only was central government level. Included affiliated ministries directly related to health (e.g., the National Health Commission), and those that could influence health-related issues (Appendix 4).

### 2.4 Search strategy

The search strategy was optimized and developed through team discussion. Policy documents in Chinese were identified using the keyword *Fen Ji Zhen Liao*. If the built-in search function did not work properly, third-party search engines^3^ were used to conduct website searches. After conducting the search, two researchers independently carried out the screening process with any discrepancies or uncertainties being resolved through group discussions with other researchers.

### 2.5 Data extraction

For each policy document, the extracted information included the title, releasing ministry, issue date, type, and policy initiatives. Based on the policy documents’ releasing ministries, we classified them into “singular releases” or “joint releases.” Regarding the release time, each policy document was further divided into four periods: 2009–2010, 2011–2015, 2016–2020 and 2021–2022. These four periods are consistent with the 11th, 12th, 13th, and 14th five-year plans of the Chinese central government.

### 2.6 Policy content analysis

**Figure 1:**
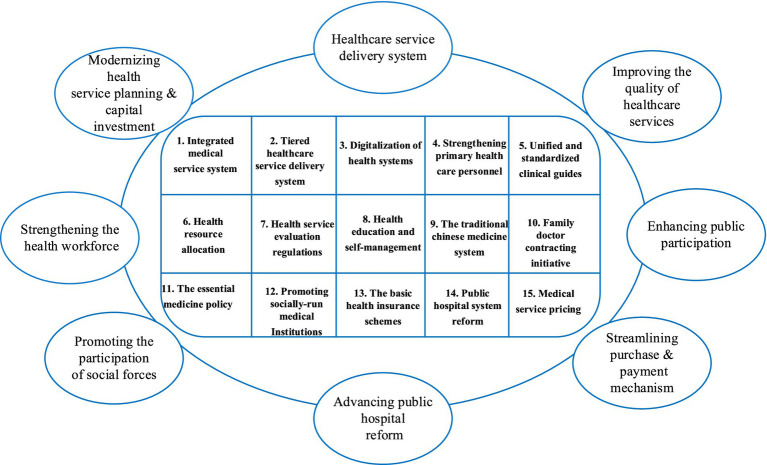
The analytical framework.

There were four steps for data synthesis. First, the essential data items of all eligible policy documents were tabulated and summarized. Second, the interrelationship of policy documents was identified from the formulation basis briefly described in the background of each policy. Third, the hierarchical medical system-related con-tent of the policy documents was coded and mapped to the analytical framework as described (Figure 1). The people-centered integrated care model was jointly proposed by the WHO, the World Bank and the Chinese government to promote the implementation of hierarchical medical system (20). Four, the policy instruments were used to conduct a quantitative analysis of the policy content (Appendix 5). All coding was performed using the NVivo 12 software for data management.

## 3 Results

**Figure 2:**
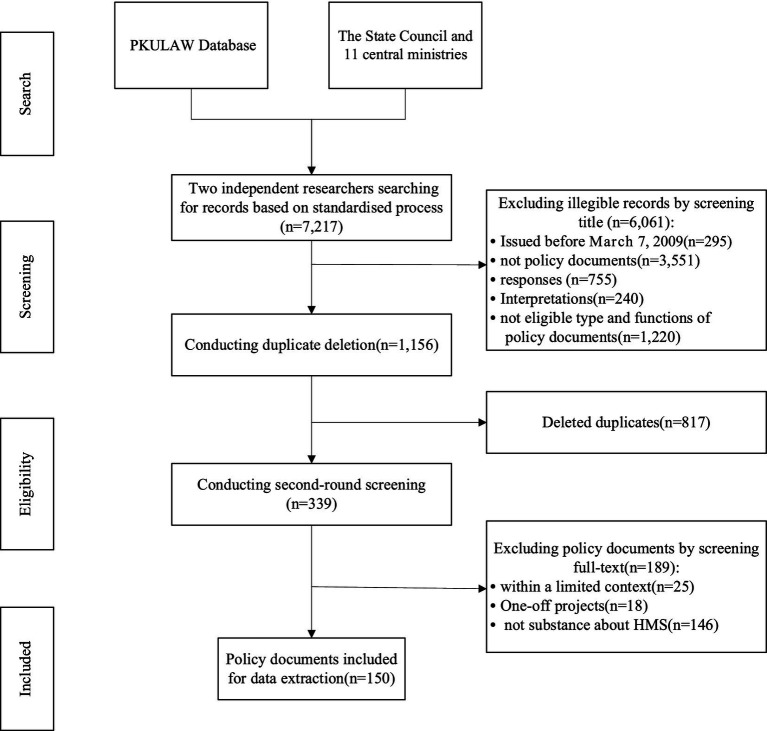
The flow diagram of policy identification.

The standard search yielded 7,217 records issued by the PKULAW Database, the Chinese State Council, and 11 affiliated ministries (Figure 2). After screening the title and the full text based on the eligibility criteria, a total of 150 policy documents were finally included in this study.

### 3.1 Volume and variety of included policies

**Figure 3:**
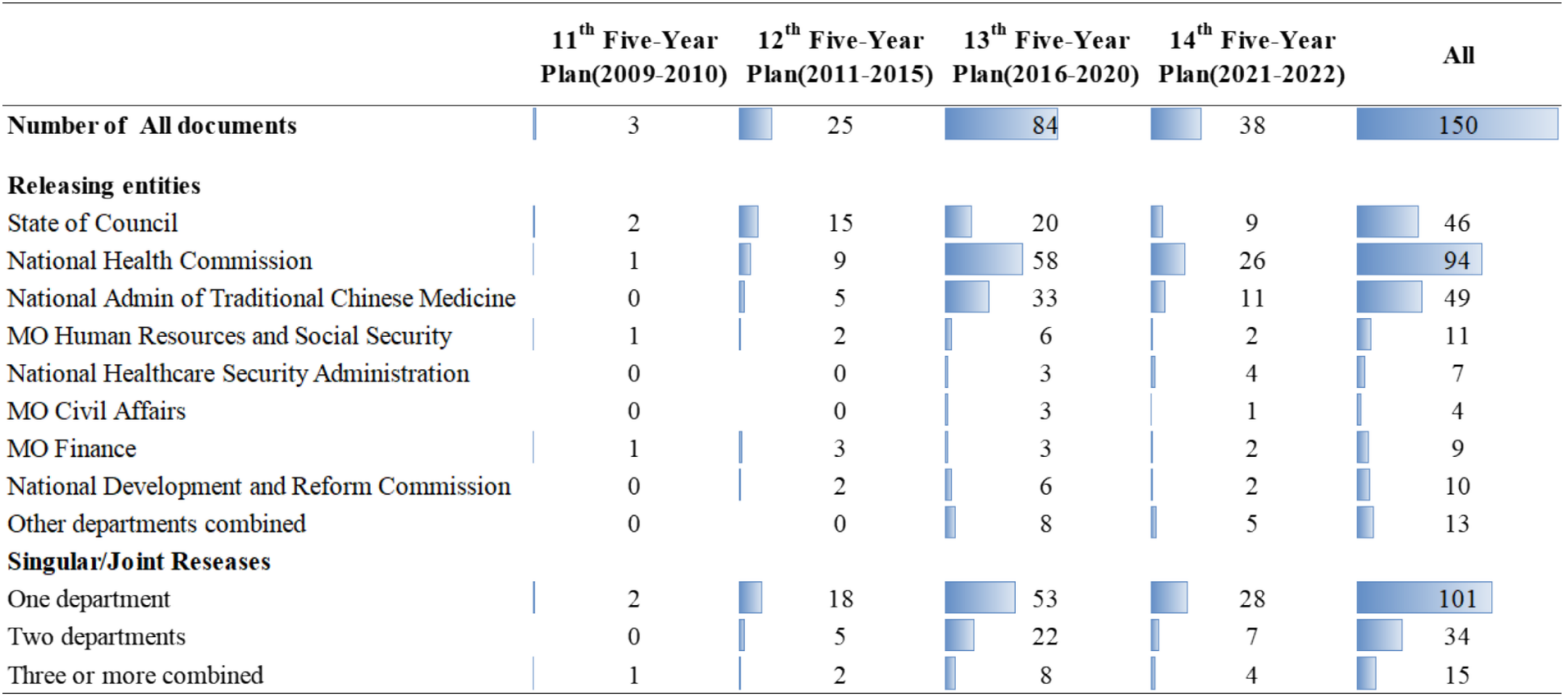
Temporal distribution of HMS policy documents. ^a^MO: Ministry Of; admin: Administration. ^b^Other ministries (*n* = 4): Ministry of Education, Ministry of Science and Technology, National Medical Products Administration, Ministry of Industry and Information Technology.

Since the 2009 health reform, the number of policies has generally shown an upward trend. About half of the eligible policy documents (*n* = 84) were released during the 13th Five-Year plan, followed by the 14th Five-Year plan (Figure 3). This was because, in 2015, the central government issued the “Guiding Opinions of the State Council on Promoting the Construction of Hierarchical Medical System,” which guided the development of policy documents at the national level. The Chinese National Health Commission issued the highest number in total and across each five-year plan (*n* = 94), followed by the Chinese State Council (*n* = 46). And 101 of the policies included were issued by a single ministry. Although the number of policies jointly developed by multiple ministries was only 49, the number of policies continued to increase during each five-year plan. And joint policy issuance has become a mainstream trend. Notably, the publication of multi-sectoral policies was still limited to a few specific sectors.

### 3.2 Multi-sectorial collaborative HMS policy making

**Figure 4:**
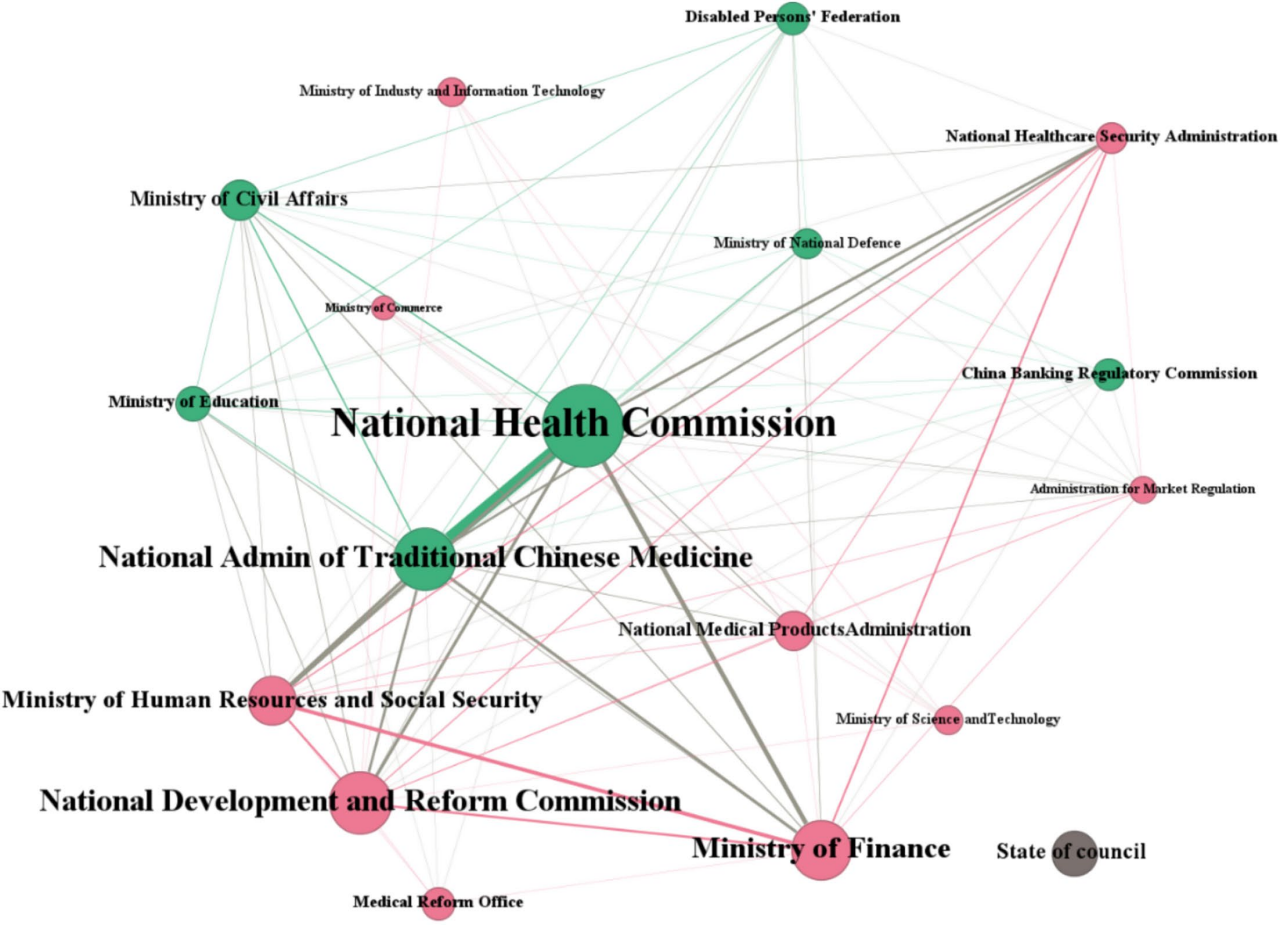
Multi-sectorial collaborative HMS policy making.

In Figure 4, circular nodes represented participating ministries, the size of the nodes reflected the relative frequency of the ministry’s involvement in policy making, while the straight line represented the common cooperative relationship between ministries. As a separate node, the State Council issued a total of 46 national policies, with the content of these policies being more closely related to major plans and outlines. Although the State Council hardly ever issued policies jointly with other affiliated ministries, the policies it did issue play a leading role in policy making bodies. In the network, the National Health Commission had the largest node, with 138 policies issued. The second largest node was the National Administration of Traditional Chinese Medicine, which issued 46 policies. The Disabled Persons’ Federation and the National Medical Products Administration had the smallest nodes and issued the least policies. The National Health Commission was at the center of the entire network and had collaborative relationships with multiple ministries, indicating that it was the major responsible affiliated department for policy making. The National Administration of Traditional Chinese Medicine, the National Development and Reform Commission, and the Ministry of Finance were also important nodes, which together with the National Health Commission constituted the core institutions of the multi-sectoral cooperation.

### 3.3 Key patterns of HMS policy making

**Figure 5:**
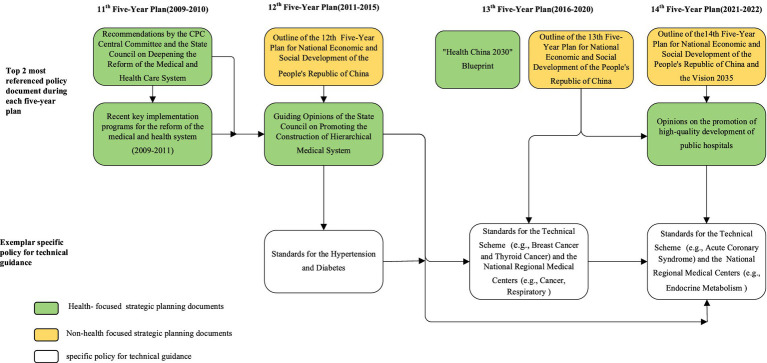
Leading HMS policies evolution and policy cross-referencing.

As shown in Figure 5, we identified the top two most referenced policy documents and specific policy guidance documents during each five-year planning period. Some of which were health-focused (e.g., the green rounded rectangle) and some were not (e.g., the orange rounded rectangle). The vertical arrows in Figure 5 represented the evolution path of national policy generation. Firstly, the Chinese central government engaged in top-level design and released high-level strategic planning documents, some of which outlined the macro goals of hierarchical medical system. In addition, these strategic planning documents, as leading documents, were frequently referenced by other policy documents. Subsequently, policies were further made and developed by the State Council or affiliated ministries with the exploration of local practice and policies innovation. Some of these policy documents described the policy objectives and contents of the hierarchical medical system in detail. For example, the “Guiding Opinions on Promoting the Construction of Hierarchical Medical System.” Finally, there were bottom-level policy documents that provided technical guidance or requirements. On the other hand, the horizontal arrows indicated a progressive policy retention and evolvement process. Some more recent policy documents were built upon and refined from historical ones, e.g., standards for the technical scheme (previous ones cross-referenced by the subsequent versions).

### 3.4 Major policy initiatives for HMS by PCIC model

**Figure 6:**
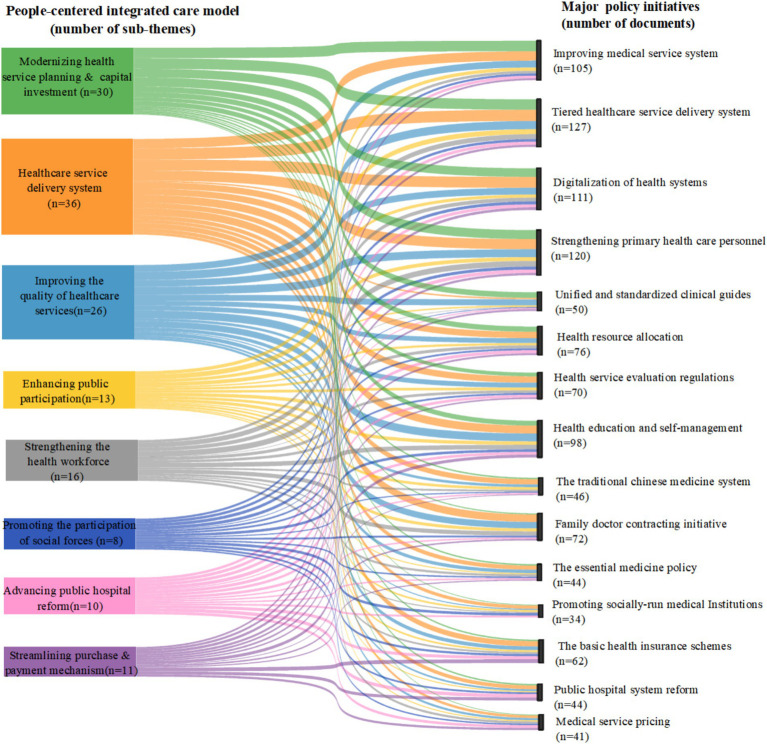
Policy Initiatives for PCIC-focused hierarchical medical system.

Through the inductive coding of the 150 policy documents, we identified the number of policy documents in each block, the number of policy initiatives, and the flow relationships between them. We found that each of the 15 major policy initiatives was mentioned in multiple policy documents (Figure 6). These policy documents covered multiple people-centered integrated care building blocks. This indicated that the 15 policy initiatives proposed by the Chinese government adhered to the principles of people-centered integrated care. The leading three building blocks were “modernizing health service planning and capital investment (*n* = 30),” “the healthcare service delivery system (*n* = 36)” and “improving the quality of healthcare services (*n* = 26).” And the top three policy initiatives were” tiered healthcare service delivery system” (*n* = 127), “strengthening primary healthcare personnel” (*n* = 120), and “digitalization of health systems” (*n* = 111).

### 3.5 Distribution of HMS policy instruments

During implementing the policy of hierarchical medical system, the central government comprehensively used three policy instruments: demand, supply and environment. The supply-side policy instruments accounted for 43.2%, demand-side policy instruments accounted for 19.5% and environmental policy instruments accounted for 37.3%. Within the supply-side policy instruments, the institution construction, resource allocation, personnel training, technical support and digitalization of health systems, respectively, accounted for 17.3, 12.7, 25.1, 18.8 and 26%. Within the demand-side policy instruments, the health insurance payment, price guidance, priority diagnosis and treatment, disease classification list and medicine regulation, respectively, accounted for 35.9, 19.9, 10.9, 18.6 and 14.7%. Within the environmental policy instruments, target planning, regulatory control, performance incentives, functional supervision and policy promotion, respectively, accounted for 18.4, 26.4, 14.7, 17.4 and 23.1%.

**Figure 7:**
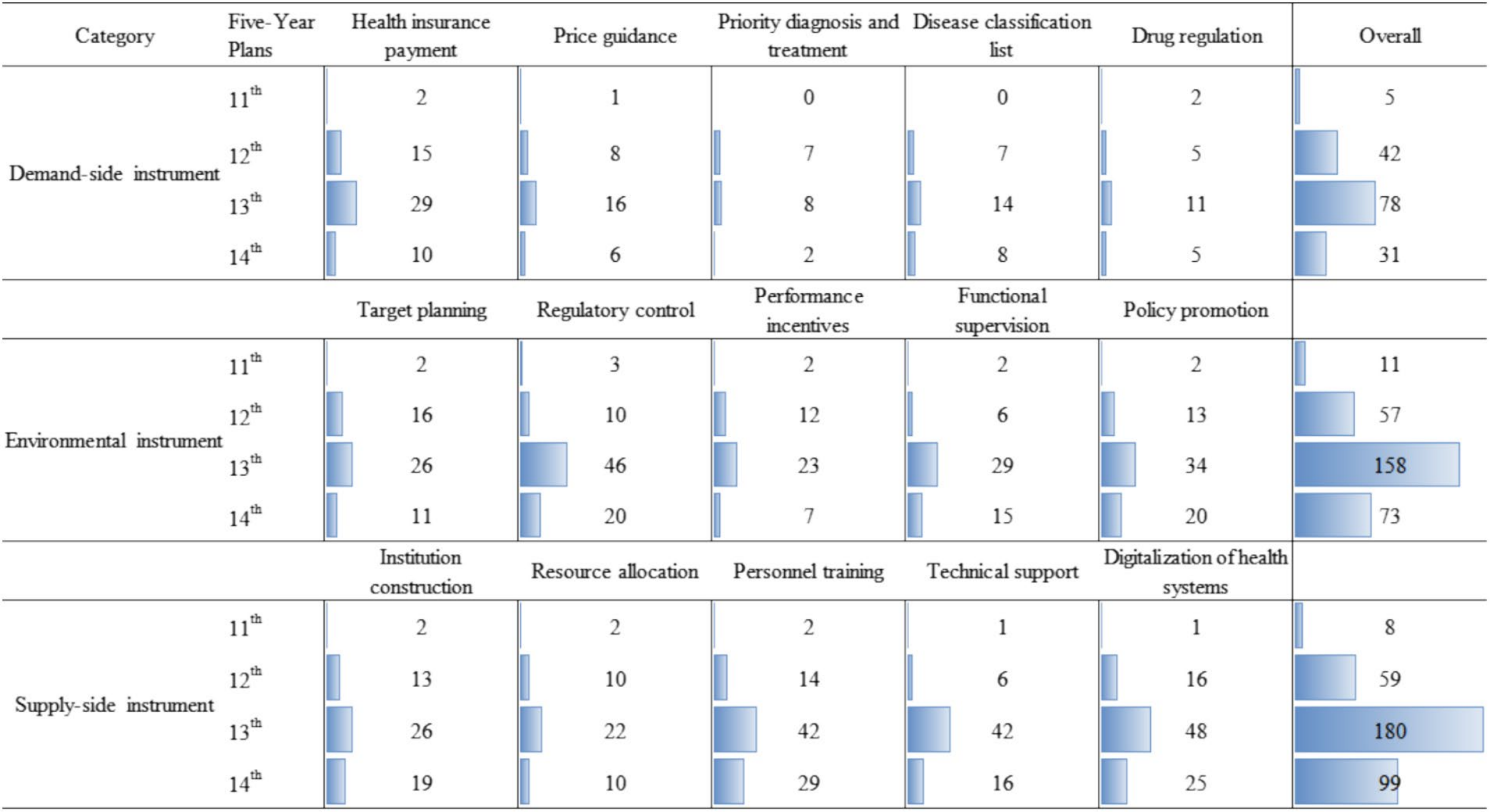
Distribution of policy instruments.

As shown in Figure 7, from 2009 to 2010, demand-side, supply-side and environmental policy instruments were used in a relatively balanced way, with 5, 8 and 11 times, respectively. From 2011 to 2015, the gap in the use of the three types of policy instruments increased. Demand-side, supply-side and environmental policy instruments were 42, 59 and 57 times, respectively. From 2016 to 2020, the gap had further widened, mainly due to the surge in the supply-side instruments and environmental instruments. From 2021 to 2023, the relationship between the three policy instruments was similar to that of the previous five-year plan.

## 4 Discussion

This review provided an in-depth overview of national policies related to the hierarchical medical system since 2009 health reform, including the distribution pattern by date and ministries of policy documents, policy evolution, 15 major policy initiatives as well as characteristics of policy instruments. These strategies and actions demonstrated coherent leadership and sustained resource support, which were critical for policy planning, implementation, and adaptive adjustments. Over the past 14 years, China has made steady progress toward universal health coverage, demonstrating China’s strong political commitment (21, 22). The period 2009–2015 was the transformation phase from concept to implementation of hierarchical medical system, and partial pilot projects were carried out in some areas. In 2015, the Chinese government issued a guideline on promoting the construction of the hierarchical medical system. In this policy, the goal of the hierarchical medical system was articulated as to create an accessible, high-quality, and affordable health care system (23). This means that the overall goal and basic tasks of the hierarchical medical system have been formed, and the system has been confirmed and promoted nationwide. About half of the policy documents were issued in the 13th Five-Year Plan, and the polices of this period had a strong influence on the current policy implementation and future policy formulation, which is also supported by the significantly increased financial investment (16).

### 4.1 Governance and intersectoral collaboration

The establishment of an integrated health information system based on the three-tier health care system had become a priority for China to improve the efficiency and accessibility of health care services. However, achieving HMS was a tough and long-term task that required multi-sectoral cooperation. The absence of sustained mechanisms for multisectoral collaboration constrains progress in health reform. Despite the collaborative vision of Healthy China 2030, fragmented governance and poor coordination between health, development, social security, and education sectors reduces the consideration of health in all policies (24). The coordination between the central and local governments was reflected in the development of local policies. The influence of top-level ministries was more macro, and the influence of local ministries was more specific and clear, thus forming a policy synergy to improve the implementation effect of local policies. In recent years, the HMS policies lack top-level design, which is not conducive to the absorption and practice of local policies. Therefore, the construction of China’s HMS was led by the State of Council, with the National Health Commission and the National Administration of Traditional Chinese Medicine as the core, and the Ministry of Human Resources, the Ministry of Finance, the National Development and Reform Commission, the National Healthcare Security Administration and other departments cooperate together to ensure mutual support and effective coordination among policies.

### 4.2 Evolution of leading HMS policies

In this review, we identified two patterns of policy making. The first pattern was a “top-down” path (from the State Council to the ministries). This was the most common pattern in China’s overall national policymaking practice, implying a strong influence of the State Council on the policymaking process. The other one was a “parallel ministry” pattern (from one ministry to another). This pattern was also clearly evident in some ministries. For example, the specific policy guidance was formulated by the relevant related ministries. Notably, the ‘bottom-up’ path also existed in this policy area. For example, the medical alliance system was a product of local policies. With the accumulation of local experience, the “medical alliance system” began to appear in national policies. Regardless of the policy making pathway, it was essential to minimize fragmentation among policies and remove barriers at all levels. This requires the Chinese government to do a good job of high-level design and policy implementation path.

### 4.3 Major policy initiatives for HMS-focused

Out of the 15 major policy initiatives for HMS, the tiered healthcare service delivery system, strengthening health workforce, and reforming the health insurance system were the most common policy priorities (25, 26). Some policies also embodied the “people-centered” principle, such as improving the quality of health services, building a harmonious relationship, promoting the coordinated service in all levels of medical institutions, pushing resources down to primary health care institutions, and improving the regional health service planning (27–29). All of these depend critically on efficient national leadership and coordinated central governance mechanism. Notably, China’s health resources are mainly concentrated in cities, and the allocation of tertiary medical resources is more prominent in cities. Therefore, the policy of hierarchical medical system was first implemented in urban areas and then transferred to rural areas. The implementation of HMS increased the possibility of urban residents going to primary medical institutions, compared with rural residents. Some potential reasons for this urban–rural difference are that rural residents distrust primary care facilities and are not satisfied with the quality of primary care, both of which hinder its effectiveness (30, 31). The government should adopt better means (e.g., patient satisfaction metrics) to change the patient flow and medical choices, and further carry out HMS reform.

### 4.4 Characteristics of China’s HMS policy instruments

A policy instrument is a means to achieve a policy goal. On the basis of optimizing the medical and health service supply system, the Chinese Government guides the behavior of both supply and demand sides, thus realizing the policy goal of hierarchical medical system (31). So, the supply-side policy instruments were most used. On the one hand, the efficiency of the whole healthcare service system is improved through resource integration and sharing. On the other hand, increase quality medical resources to improve the service capacity of primary health care institutions. The large gap of general practitioners and the insufficient service capacity of primary health care facilities was the main problem limiting the hierarchical medical system (32). Demand-side policy instruments were used least, resulting in a lack of direct motivation for policy development. This may be because the implementation of hierarchical medical system is closely linked to healthcare infrastructure, compelling governments to prioritize efficiency when selecting policy instruments. In order to play the incentive role of demand-oriented instruments in reducing economic burden, for key populations and common diseases, the government could increase the degree of buyer compliance through health insurance payment and price guidance instruments, and increase the degree of policy support through medical science popularization and policy promotion. In addition, a stable external environment was the basis for the development of a hierarchical medical system. Open and transparent medical functional supervision and performance were able to effectively guide the behavior of medical service providers to meet the requirements of the hierarchical medical system. In particular, China’s target planning was visionary and comprehensive, but they were often vague, resulting in weak local implementation. Training professional implementation officials, developing better operational instructions, creating policy enabling conditions, and setting more realistic timelines would be more conducive to policy implementation (16).

In summary, we found that the hierarchical medical system has always been at the core of healthcare reform, playing a crucial role in the adjusting healthcare resources, alleviating supply–demand imbalances, and addressing major obstacles to access to healthcare for the public (33). In the past years, China has largely established a hierarchical medical system model focused on a family doctor contracting system, medical service integration and health insurance reform (34, 35). However, despite notable achievements in recent years, challenges persist, including significant resistance, slow progress, and difficulties in enhancing primary medical services (33). At the same time, several limitations in this study should be mentioned. First, this paper only accessed open-sourced policy documents on government websites and some potential non-public policy documents had not been identified. Second, this paper focuses on central-level policies while neglecting possible local policy innovations in the hierarchical medical system. Third, the data retrieved in this paper was inadequate to assess the implementation and impact of these policies.

## 5 Conclusion

China is increasingly embracing the opportunities and challenges in the hierarchical medical system. However, there exist major gaps in the shortage of inter-agency communication, inadequate collaboration between regulation of medical system and service delivery, underdeveloped public hospital reform, and insufficient policies addressing demand-side policy instruments. By introducing a scoping review-based approach, we finally summarize the future directions for policy improvement. First, a unified policy framework is necessary to coordinate the development and regulation of hierarchical medical system and facilitate multi-sectoral cooperation. Second, in-depth reforms in the key regions in hierarchical medical system, such as public hospital reform, medical insurance payment. Third, balance the use of various policy instruments, giving full consideration to the characteristics of hierarchical medical system at different stages.

## Footnotes

^1^ https://osf.io/3jcvp

^2^ https://www.pkulaw.com/

^3^ https://cn.bing.com/?mkt=zh-CN
