# Supplementary material for: A scoping review of national policies for hierarchical medical system in China since the 2009 health reform
Source: Front Public Health. 2025 Jul 9;13:1606842. doi: 10.3389/fpubh.2025.1606842 (PMC12283634; doi:10.3389/fpubh.2025.1606842)
Supplement: Supplementary file 1 [file Table_1.pdf]

Appendixes

NO.	Contents	Pages
Appendix 1	Scoping Reviews (PRISMA-ScR) Checklist	1
Appendix 2	The types and functions of the policy document in China	3
Appendix 3	Questions used to facilitate the policy identification in the search of websites	4
Appendix 4	The policy source for Chinese literature	4
Appendix 5	The essential policy instrument items of all eligible policies	5

Appendix 1

Preferred Reporting Items for Systematic reviews and Meta-Analyses extension for Scoping Reviews (PRISMA-ScR) Checklist

SECTION	ITEM	PRISMA-ScR CHECKLIST ITEM	REPORTED ON PAGE #
**TITLE**			
Title	1	Identify the report as a scoping review.	1
**ABSTRACT**			
Structured summary	2	Provide a structured summary that includes (as applicable): background, objectives, eligibility criteria, sources of evidence, charting methods, results, and conclusions that relate to the review questions and objectives.	1
**INTRODUCTION**			
Rationale	3	Describe the rationale for the review in the context of what is already known. Explain why the review questions/objectives lend themselves to a scoping review approach.	2-3
Objectives	4	Provide an explicit statement of the questions and objectives being addressed with reference to their key elements (e.g., population or participants, concepts, and context) or other relevant key elements used to conceptualize the review questions and/or objectives.	2-3
**METHODS**			
Protocol and registration	5	Indicate whether a review protocol exists; state if and where it can be accessed (e.g., a Web address); and if available, provide registration information, including the registration number.	2
Eligibility criteria	6	Specify characteristics of the sources of evidence used as eligibility criteria (e.g., years considered, language, and publication status), and provide a rationale.	2
Information sources	7	Describe all information sources in the search (e.g., databases with dates of coverage and contact with authors to identify additional sources), as well as the date the most recent search was executed.	2-3
Search	8	Present the full electronic search strategy for at least 1 database, including any limits used, such that it could be repeated.	3
Selection of sources of evidence	9	State the process for selecting sources of evidence (i.e., screening and eligibility) included in the scoping review.	2
Data charting process	10	Describe the methods of charting data from the included sources of evidence (e.g., calibrated forms or forms that have been tested by the team before their use, and whether data charting was done independently or in duplicate) and any processes for obtaining and confirming data from investigators.	3
Data items	11	List and define all variables for which data were sought and any assumptions and simplifications made.	2-3
Critical appraisal of individual sources of evidence	12	If done, provide a rationale for conducting a critical appraisal of included sources of evidence; describe the methods used and how this information was used in any data synthesis (if appropriate).	Not applicable
Synthesis of results	13	Describe the methods of handling and summarizing the data that were charted.	3

SECTION	ITEM	PRISMA-ScR CHECKLIST ITEM	REPORTED ON PAGE #
**RESULTS**			
Selection of sources of evidence	14	Give numbers of sources of evidence screened, assessed for eligibility, and included in the review, with reasons for exclusions at each stage, ideally using a flow diagram.	3-4
Characteristics of sources of evidence	15	For each source of evidence, present characteristics for which data were charted and provide the citations.	2-3
Critical appraisal within sources of evidence	16	If done, present data on critical appraisal of included sources of evidence (see item 12).	Not applicable
Results of individual sources of evidence	17	For each included source of evidence, present the relevant data that were charted that relate to the review questions and objectives.	Not applicable
Synthesis of results	18	Summarize and/or present the charting results as they relate to the review questions and objectives.	3-7
**DISCUSSION**			
Summary of evidence	19	Summarize the main results (including an overview of concepts, themes, and types of evidence available), link to the review questions and objectives, and consider the relevance to key groups.	7-9
Limitations	20	Discuss the limitations of the scoping review process.	9
Conclusions	21	Provide a general interpretation of the results with respect to the review questions and objectives, as well as potential implications and/or next steps.	9
**FUNDING**			
Funding	22	Describe sources of funding for the included sources of evidence, as well as sources of funding for the scoping review. Describe the role of the funders of the scoping review.	9

JBI = Joanna Briggs Institute; PRISMA-ScR = Preferred Reporting Items for Systematic reviews and Meta-Analyses extension for Scoping Reviews.

From: Tricco AC, Lillie E, Zarin W, O'Brien KK, Colquhoun H, Levac D, et al. PRISMA Extension for Scoping Reviews (PRISMAScR): Checklist and Explanation. Ann Intern Med. 2018;169:467–473. doi: 10.7326/M18-0850.

Appendix 2

The types and functions of the policy document in China

Types (in Chinese)	Function description	Transmission direction	Advice strength	Formulation purpose	Included
Resolution (决议)	1. The authority announces major decisions approved at the conference	From higher authority to lower authority	Command/ Inform	Work deployment	Yes
Decision (决定)	1. The authority makes decisions for important issues	From higher authority to lower authority	Command	Goal planning /Work deployment	Yes
	2. The authority rewards and punishes relevant departments or people	From higher authority to lower authority	Command	Fact statement	No
	3. The authority changes or revokes inappropriate decisions from lower authorities	From higher authority to lower authority	Command	Fact statement	No
Order (命令/令)	1. The authority issues administrative regulations and rules	From higher authority to lower authority	Command	Goal planning/Work deployment	Yes
	2. The authority announces the implementation of mandatory measures	From higher authority to lower authority	Command	Goal planning/Work deployment	Yes
	3. The authority approves the award and promotion	From higher authority to lower authority	Command	Fact statement	No
	4. The authority rewards relevant departments or people	From higher authority to lower authority	Command	Fact statement	No
Bulletin (公报)	1. The authority announces important meetings	From higher authority to lower authority	Inform	Fact statement	No
	2. The authority announces important events (including diplomatic activities or macro statistics)	From higher authority to lower authority	Inform	Fact statement	No
Announcement (公告)	1.The authority announces important or statutory matters to domestic and foreign institutions	From higher authority to lower authority	Command/ Inform	Work deployment	Yes
				Fact statement	No
Note (通告)	1.The authority announces matters that should be complied with or known to the public within jurisdiction	From higher authority to lower authority	Command/ Suggestion	Goal planning/Work deployment	Yes
				Fact statement	No
Opinion (意见)	1. The authority elaborates insights and solutions to important issues	From higher authority to lower authority	Command/ Suggestion	Goal planning/Work deployment/ Opinions communication	Yes
Notice (通知)	1. The authority publishes or communicate matters that are required to be known or implemented by lower or parallel authorities	From higher authority to lower authority/ at the same level	Command	Goal planning/Work deployment/ Opinions communication	Yes
	2. The authority approves or forwards official documents from lower or parallel authorities	From higher authority to lower authority/ at the same level	Command	Goal planning/Work deployment/ Opinions communication	Yes
Notification (通报)	1. The authority praises certain advanced departments or people	From higher authority to lower authority	Command	Fact statement	No
	2. The authority criticizes certain advanced departments or people	From higher authority to lower authority	Command	Fact statement	No
	3. The authority conveys important spirit and inform important information.	From higher authority to lower authority	Command/ Suggestion	Goal planning/Work deployment/ Opinions communication	Yes
Report (报告)	1.The lower authority reports the work or reflect the situation to higher authorities, and replies to the inquiry of higher authorities	From lower authority to higher authority	Inform/ Discussion or Deliberation	Opinions communication	No
Request (请示)	1. The lower authority requests instructions and approval from higher authorities	From lower authority to higher authority	Discussion or Deliberation	Opinions communication	No
Reply (批复)	1. The higher authority responds to requests from higher authorities	From higher authority to lower authority	Suggestion/ Inform	Opinions communication/ Fact statement	No
Motion (议案)	1. People's governments at all levels submit matters to the people's congresses at the same level or the standing committees of the people's congresses in accordance with legal procedures	At the same level	Discussion or Deliberation	Opinions communication	No
Letter (函)	1. Non -subordinate departments negotiate work, ask and answer questions, request approval, and reply to approval matters	At the same level	Inform/Discussion or Deliberation	Opinions communication	No
Minutes (纪要)	1. The authority records the main situation and agreed matters of the meeting	From lower authority to higher authority/at the same level	Inform	Fact statement	No

Appendix 3

These questions used to facilitate the policy identification in the search of websites

Q1 Is this record a policy document?

Yes -> go to Q2

No -> excluded

Q2 Is this policy document published?

Yes -> go to Q3

No -> excluded

Q3 Is the policy published between 2009 and 2022?

Yes -> go to Q4

No -> excluded

Q4Is policy document issued by the State Council and its affiliated departments?

Yes -> go to Q5

No -> excluded

Q5 Is the type and function of policy document eligible?

Yes -> go to Q6

No -> excluded

Q6 Does this policy explicitly include the keyword focusing to HMS?

Yes -> go to Q7

No -> excluded

Q7 Is the full text of policy available?

Yes -> go to Q8

No -> excluded

Q8 Is the policy document limited within a limited context?

Yes -> excluded

No -> Included

Appendix 4

The policy source for Chinese literature

ID	Name in English	Nature of source	URL of website
1	PKULAW – Legal Information Database	Chinese website	https://www.pkulaw.com/
2	State Council	The State Council	http://www.gov.cn/guowuyuan/
3	General Office of the State Council	General Office of the State Council	http://www.gov.cn/guowuyuan/
4	National Development and Reform Commission	Departments of the State Council	http://www.ndrc.gov.cn/
5	Ministry of Education	Departments of the State Council	http://www.moe.gov.cn/
6	Ministry of Science and Technology	Departments of the State Council	http://www.most.gov.cn/
7	Ministry of Civil Affairs	Departments of the State Council	http://www.mca.gov.cn/
8	Ministry of Finance	Departments of the State Council	http://www.mof.gov.cn/index.htm
9	Ministry of Human Resources and Social Security	Departments of the State Council	http://www.mohrss.gov.cn/
10	National Health Commission	Departments of the State Council	http://www.nhc.gov.cn/
11	National Healthcare Security Administration	Agencies directly under the State Council	http://www.nhsa.gov.cn/
12	National Administration of Traditional Chinese Medicine	State Bureau under the Administration of Ministries and Committees of the State Council	http://www.satcm.gov.cn
13	National Medical Products Administration	State Bureau under the Administration of Ministries and Committees of the State Council	http://www.nmpa.gov.cn /WS04/CL2042/
14	Ministry of Industry and Information Technology	Departments of the State Council	www.miit.gov.cn

Appendix 5

The essential policy instrument items of all eligible policies

Policy Instruments	Secondary Instruments	Number	Meaning
Supply-side instruments	Institution construction	60	Enhancing the infrastructure construction of medical institutions, improving the healthcare service network, and enhancing service capabilities
	Resource allocation	44	Allocation of resources such as bed capacity and financial subsidies based on the functional adjustment of medical institutions
	Personnel training	87	Strengthening the construction of primary healthcare personnel through various forms of education and training
	Technical support	65	Providing technical support and mentorship from higher-level medical institutions to lower-level ones
	Digitalization of health systems	90	Establishing a medical information sharing platform to facilitate the vertical flow of medical resources through digital means
Demand-side instruments	Healthcare insurance payment	56	Using differentiated payment through medical insurance to guide patients in seeking medical treatment in an orderly manner
	Price guidance	31	Setting differentiated medical service prices based on the level of healthcare institutions to guide patients in seeking medical treatment rationally
	Priority diagnosis and treatment	17	Guiding community members to seek initial medical consultation at the grassroots level by prioritizing referrals and hospital admissions for primary care providers
	Disease classification list	29	Establishing referral guidelines and standardizing the referral process for healthcare institutions at all levels. Implementing standardized diagnosis and treatment protocols to ensure consistency and quality of medical care
	Drug regulation	23	Expanding the variety and quantity of drugs available at primary healthcare institutions, enhancing their connection with higher-level institutions, and extending the time limit for drug dispensing at primary healthcare facilities
Environmental instruments	Target planning	55	Overall planning of regional healthcare resources
	Regulatory control	79	To regulate the behavior of all parties involved in healthcare services, various regulations and institutional measures can be implemented
	Performance incentives	44	Establish a performance-based compensation and fiscal compensation mechanism
	Functional supervision	52	Regulating the functions and service quality of healthcare institutions
	Policy promotion	69	Widely publicizing the policy to the target audience
